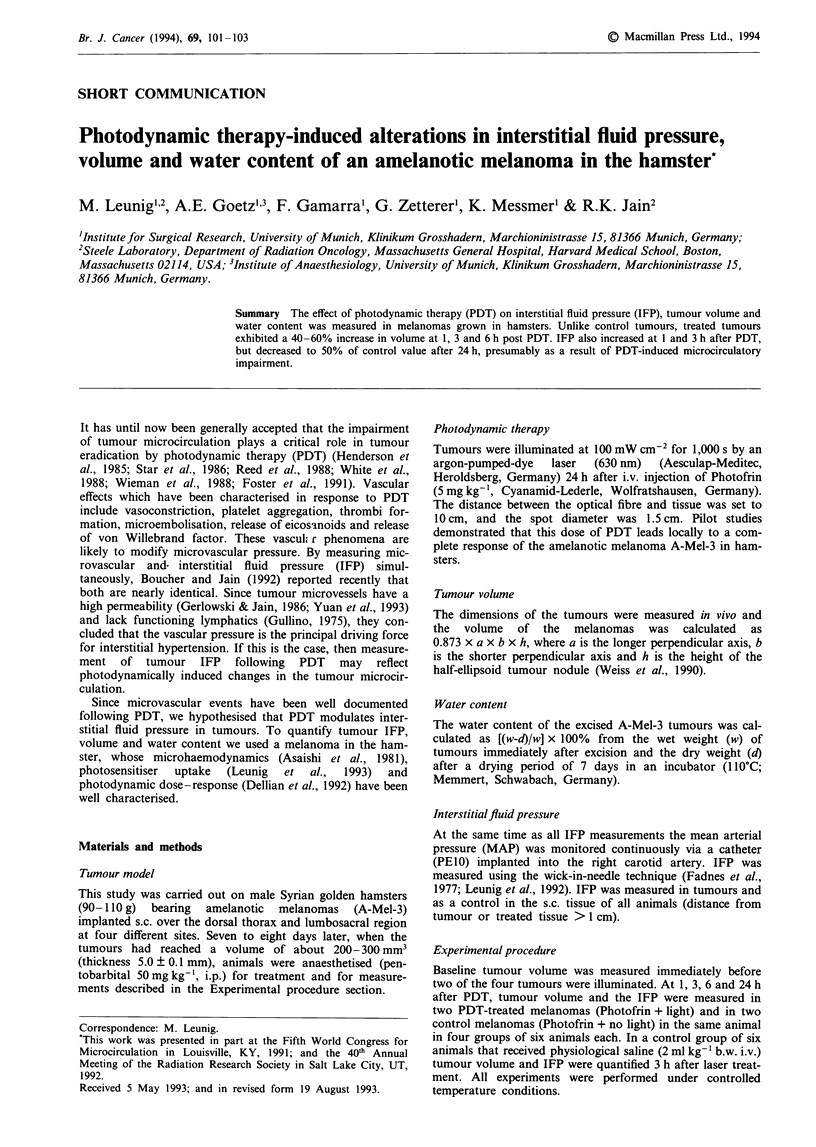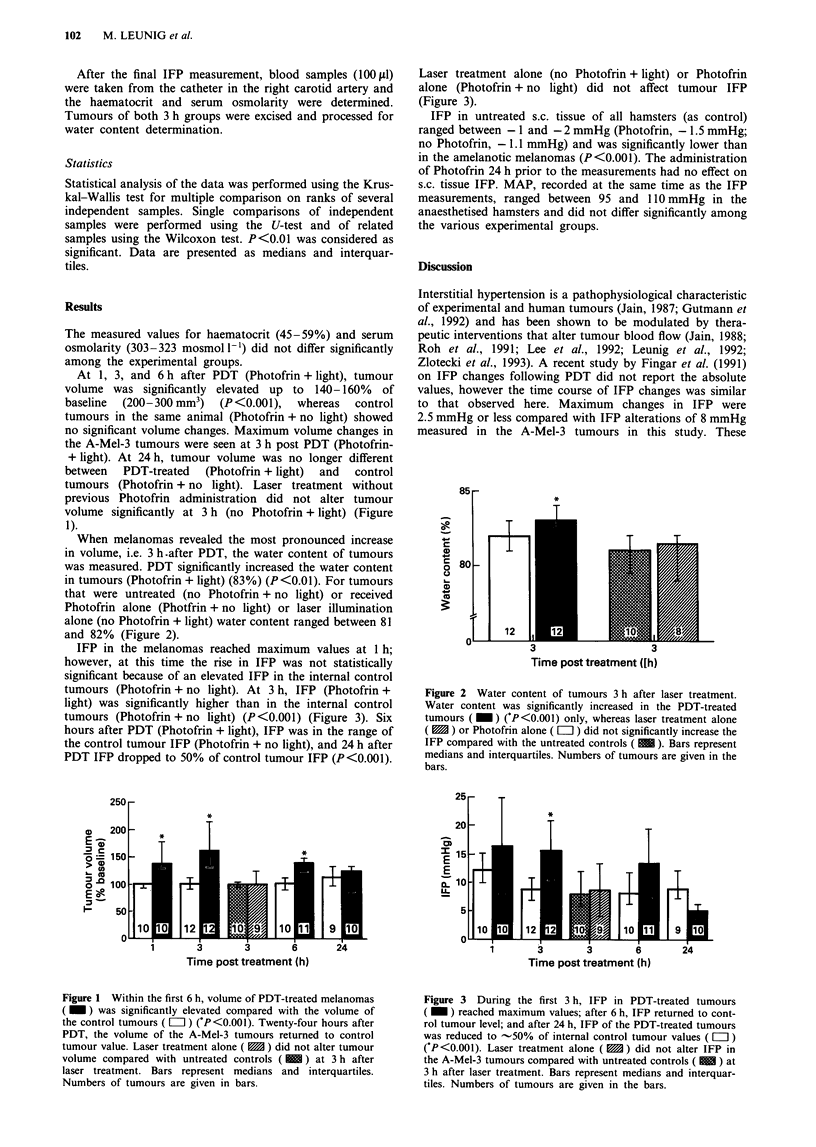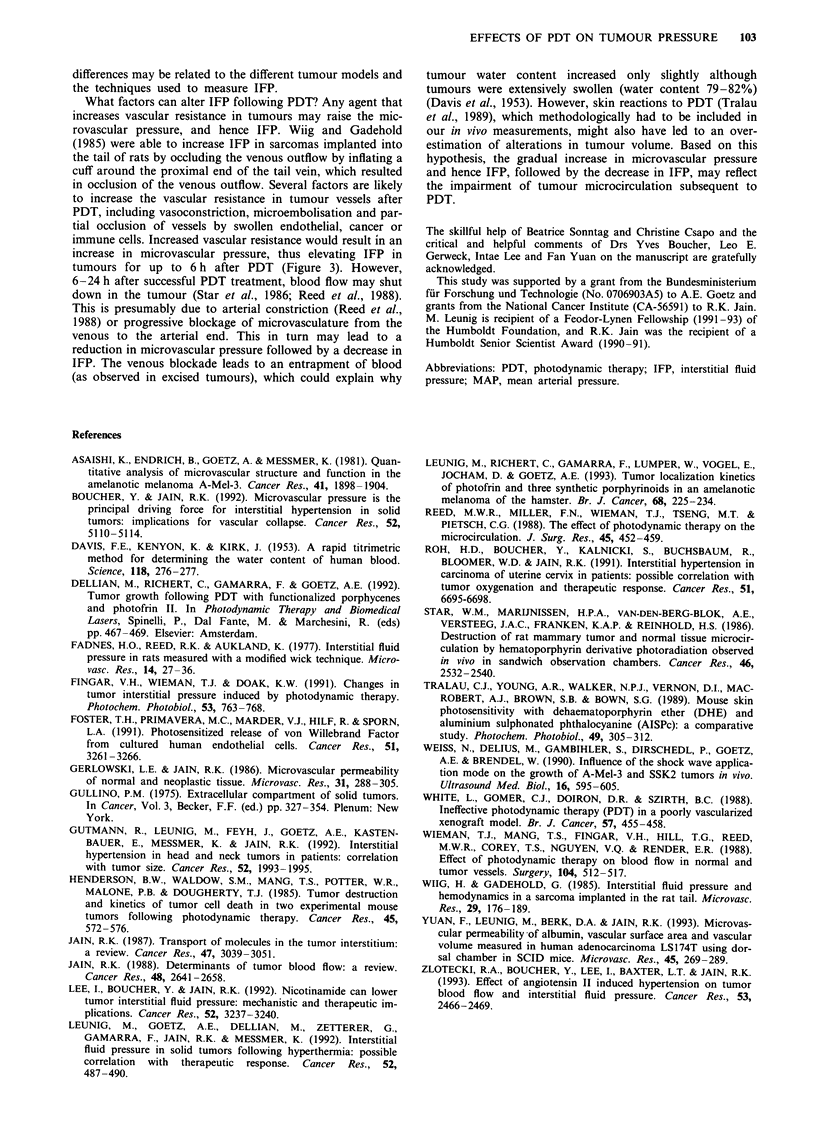# Photodynamic therapy-induced alterations in interstitial fluid pressure, volume and water content of an amelanotic melanoma in the hamster.

**DOI:** 10.1038/bjc.1994.15

**Published:** 1994-01

**Authors:** M. Leunig, A. E. Goetz, F. Gamarra, G. Zetterer, K. Messmer, R. K. Jain

**Affiliations:** Institute for Surgical Research, University of Munich, Klinikum Grosshadern, Germany.

## Abstract

The effect of photodynamic therapy (PDT) on interstitial fluid pressure (IFP), tumour volume and water content was measured in melanomas grown in hamsters. Unlike control tumours, treated tumours exhibited a 40-60% increase in volume at 1, 3 and 6 h post PDT. IFP also increased at 1 and 3 h after PDT, but decreased to 50% of control value after 24 h, presumably as a result of PDT-induced microcirculatory impairment.


 © Macmillan Press Ltd., 1994

SHORT COMMUNICATION

### Photodynamic therapy-induced alterations in interstitial fluid pressure, volume and water content of an amelanotic melanoma in the hamster*

M. Leunig[1,2], A.E. Goetz[1,3], F. Gamarra[1], G. Zetterer[1], K. Messmer[1] & R.K. Jain[2]

[1]*Institute for Surgical Research, University of Munich, Klinikum Grosshadern, Marchioninistrasse 15, 81366 Munich, Germany;* [2]*Steele Laboratory, Department of Radiation Oncology, Massachusetts General Hospital, Harvard Medical School, Boston, Massachusetts 02114, USA;* [3]*Institute of Anaesthesiology, University of Munich, Klinikum Grosshadern, Marchioninistrasse 15, 81366 Munich, Germany.*

**Summary** The effect of photodynamic therapy (PDT) on interstitial fluid pressure (IFP), tumour volume and water content was measured in melanomas grown in hamsters. Unlike control tumours, treated tumours exhibited a 40-60% increase in volume at 1, 3 and 6 h post PDT. IFP also increased at 1 and 3 h after PDT, but decreased to 50% of control value after 24 h, presumably as a result of PDT-induced microcirculatory impairment.

It has until now been generally accepted that the impairment of tumour microcirculation plays a critical role in tumour eradication by photodynamic therapy (PDT) (Henderson *et al.*, 1985; Star *et al.*, 1986; Reed *et al.*, 1988; White *et al.*, 1988; Wieman *et al.*, 1988; Foster *et al.*, 1991). Vascular effects which have been characterised in response to PDT include vasoconstriction, platelet aggregation, thrombi formation, microembolisation, release of eicosanoids and release of von Willebrand factor. These vascular phenomena are likely to modify microvascular pressure. By measuring microvascular and interstitial fluid pressure (IFP) simultaneously, Boucher and Jain (1992) reported recently that both are nearly identical. Since tumour microvessels have a high permeability (Gerlowski & Jain, 1986; Yuan *et al.*, 1993) and lack functioning lymphatics (Gullino, 1975), they concluded that the vascular pressure is the principal driving force for interstitial hypertension. If this is the case, then measurement of tumour IFP following PDT may reflect photodynamically induced changes in the tumour microcirculation.

Since microvascular events have been well documented following PDT, we hypothesised that PDT modulates interstitial fluid pressure in tumours. To quantify tumour IFP, volume and water content we used a melanoma in the hamster, whose microhaemodynamics (Asaishi *et al.*, 1981), photosensitiser uptake (Leunig *et al.*, 1993) and photodynamic dose-response (Dellian *et al.*, 1992) have been well characterised.

### Materials and methods

### *Tumour model*

This study was carried out on male Syrian golden hamsters (90-110 g) bearing amelanotic melanomas (A-Mel-3) implanted s.c. over the dorsal thorax and lumbosacral region at four different sites. Seven to eight days later, when the tumours had reached a volume of about 200-300 $mm^3$ (thickness 5.0 ± 0.1 mm), animals were anaesthetised (pentobarbital 50 mg $kg^{-1}$, i.p.) for treatment and for measurements described in the Experimental procedure section.

### *Photodynamic therapy*

Tumours were illuminated at 100 mW $cm^{-2}$ for 1,000 s by an argon-pumped-dye laser (630 nm) (Aesculap-Meditec, Heroldsberg, Germany) 24 h after i.v. injection of Photofrin (5 mg $kg^{-1}$, Cyanamid-Lederle, Wolfratshausen, Germany). The distance between the optical fibre and tissue was set to 10 cm, and the spot diameter was 1.5 cm. Pilot studies demonstrated that this dose of PDT leads locally to a complete response of the amelanotic melanoma A-Mel-3 in hamsters.

### *Tumour volume*

The dimensions of the tumours were measured *in vivo* and the volume of the melanomas was calculated as $0.873 \times a \times b \times h$, where $a$ is the longer perpendicular axis, $b$ is the shorter perpendicular axis and $h$ is the height of the half-ellipsoid tumour nodule (Weiss *et al.*, 1990).

### *Water content*

The water content of the excised A-Mel-3 tumours was calculated as $[(w-d)/w] \times 100\%$ from the wet weight ($w$) of tumours immediately after excision and the dry weight ($d$) after a drying period of 7 days in an incubator (110°C; Memmert, Schwabach, Germany).

### *Interstitial fluid pressure*

At the same time as all IFP measurements the mean arterial pressure (MAP) was monitored continuously via a catheter (PE10) implanted into the right carotid artery. IFP was measured using the wick-in-needle technique (Fadnes *et al.*, 1977; Leunig *et al.*, 1992). IFP was measured in tumours and as a control in the s.c. tissue of all animals (distance from tumour or treated tissue > 1 cm).

### *Experimental procedure*

Baseline tumour volume was measured immediately before two of the four tumours were illuminated. At 1, 3, 6 and 24 h after PDT, tumour volume and the IFP were measured in two PDT-treated melanomas (Photofrin + light) and in two control melanomas (Photofrin + no light) in the same animal in four groups of six animals each. In a control group of six animals that received physiological saline (2 ml $kg^{-1}$ b.w. i.v.) tumour volume and IFP were quantified 3 h after laser treatment. All experiments were performed under controlled temperature conditions.

Correspondence: M. Leunig.
*This work was presented in part at the Fifth World Congress for Microcirculation in Louisville, KY, 1991; and the 40th Annual Meeting of the Radiation Research Society in Salt Lake City, UT, 1992.
Received 5 May 1993; and in revised form 19 August 1993.

After the final IFP measurement, blood samples (100 µl) were taken from the catheter in the right carotid artery and the haematocrit and serum osmolarity were determined. Tumours of both 3 h groups were excised and processed for water content determination.

### *Statistics*

Statistical analysis of the data was performed using the Kruskal–Wallis test for multiple comparison on ranks of several independent samples. Single comparisons of independent samples were performed using the *U*-test and of related samples using the Wilcoxon test. $P<0.01$ was considered as significant. Data are presented as medians and interquartiles.

### Results

The measured values for haematocrit (45–59%) and serum osmolarity (303–323 mosmol $l^{-1}$) did not differ significantly among the experimental groups.

At 1, 3, and 6 h after PDT (Photofrin + light), tumour volume was significantly elevated up to 140–160% of baseline (200–300 $mm^3$) ($P<0.001$), whereas control tumours in the same animal (Photofrin + no light) showed no significant volume changes. Maximum volume changes in the A-Mel-3 tumours were seen at 3 h post PDT (Photofrin-+ light). At 24 h, tumour volume was no longer different between PDT-treated (Photofrin + light) and control tumours (Photofrin + no light). Laser treatment without previous Photofrin administration did not alter tumour volume significantly at 3 h (no Photofrin + light) (Figure 1).

When melanomas revealed the most pronounced increase in volume, i.e. 3 h after PDT, the water content of tumours was measured. PDT significantly increased the water content in tumours (Photofrin + light) (83%) ($P<0.01$). For tumours that were untreated (no Photofrin + no light) or received Photofrin alone (Photfrin + no light) or laser illumination alone (no Photofrin + light) water content ranged between 81 and 82% (Figure 2).

IFP in the melanomas reached maximum values at 1 h; however, at this time the rise in IFP was not statistically significant because of an elevated IFP in the internal control tumours (Photofrin + no light). At 3 h, IFP (Photofrin + light) was significantly higher than in the internal control tumours (Photofrin + no light) ($P<0.001$) (Figure 3). Six hours after PDT (Photofrin + light), IFP was in the range of the control tumour IFP (Photofrin + no light), and 24 h after PDT IFP dropped to 50% of control tumour IFP ($P<0.001$).

Laser treatment alone (no Photofrin + light) or Photofrin alone (Photofrin + no light) did not affect tumour IFP (Figure 3).

IFP in untreated s.c. tissue of all hamsters (as control) ranged between −1 and −2 mmHg (Photofrin, −1.5 mmHg; no Photofrin, −1.1 mmHg) and was significantly lower than in the amelanotic melanomas ($P<0.001$). The administration of Photofrin 24 h prior to the measurements had no effect on s.c. tissue IFP. MAP, recorded at the same time as the IFP measurements, ranged between 95 and 110 mmHg in the anaesthetised hamsters and did not differ significantly among the various experimental groups.

### Discussion

Interstitial hypertension is a pathophysiological characteristic of experimental and human tumours (Jain, 1987; Gutmann *et al.*, 1992) and has been shown to be modulated by therapeutic interventions that alter tumour blood flow (Jain, 1988; Roh *et al.*, 1991; Lee *et al.*, 1992; Leunig *et al.*, 1992; Zlotecki *et al.*, 1993). A recent study by Fingar *et al.* (1991) on IFP changes following PDT did not report the absolute values, however the time course of IFP changes was similar to that observed here. Maximum changes in IFP were 2.5 mmHg or less compared with IFP alterations of 8 mmHg measured in the A-Mel-3 tumours in this study. These

**Figure 2** Water content of tumours 3 h after laser treatment. Water content was significantly increased in the PDT-treated tumours ( ■ ) (*$P<0.001$) only, whereas laser treatment alone ( ▨ ) or Photofrin alone ( □ ) did not significantly increase the IFP compared with the untreated controls ( ▩ ). Bars represent medians and interquartiles. Numbers of tumours are given in the bars.

**Figure 1** Within the first 6 h, volume of PDT-treated melanomas ( ■ ) was significantly elevated compared with the volume of the control tumours ( □ ) (*$P<0.001$). Twenty-four hours after PDT, the volume of the A-Mel-3 tumours returned to control tumour value. Laser treatment alone ( ▨ ) did not alter tumour volume compared with untreated controls ( ▩ ) at 3 h after laser treatment. Bars represent medians and interquartiles. Numbers of tumours are given in bars.

**Figure 3** During the first 3 h, IFP in PDT-treated tumours ( ■ ) reached maximum values; after 6 h, IFP returned to control tumour level; and after 24 h, IFP of the PDT-treated tumours was reduced to ~50% of internal control tumour values ( □ ) (*$P<0.001$). Laser treatment alone ( ▨ ) did not alter IFP in the A-Mel-3 tumours compared with untreated controls ( ▩ ) at 3 h after laser treatment. Bars represent medians and interquartiles. Numbers of tumours are given in the bars.

differences may be related to the different tumour models and the techniques used to measure IFP.

What factors can alter IFP following PDT? Any agent that increases vascular resistance in tumours may raise the microvascular pressure, and hence IFP. Wiig and Gadehold (1985) were able to increase IFP in sarcomas implanted into the tail of rats by occluding the venous outflow by inflating a cuff around the proximal end of the tail vein, which resulted in occlusion of the venous outflow. Several factors are likely to increase the vascular resistance in tumour vessels after PDT, including vasoconstriction, microembolisation and partial occlusion of vessels by swollen endothelial, cancer or immune cells. Increased vascular resistance would result in an increase in microvascular pressure, thus elevating IFP in tumours for up to 6 h after PDT (Figure 3). However, 6–24 h after successful PDT treatment, blood flow may shut down in the tumour (Star *et al.*, 1986; Reed *et al.*, 1988). This is presumably due to arterial constriction (Reed *et al.*, 1988) or progressive blockage of microvasculature from the venous to the arterial end. This in turn may lead to a reduction in microvascular pressure followed by a decrease in IFP. The venous blockade leads to an entrapment of blood (as observed in excised tumours), which could explain why tumour water content increased only slightly although tumours were extensively swollen (water content 79–82%) (Davis *et al.*, 1953). However, skin reactions to PDT (Tralau *et al.*, 1989), which methodologically had to be included in our *in vivo* measurements, might also have led to an overestimation of alterations in tumour volume. Based on this hypothesis, the gradual increase in microvascular pressure and hence IFP, followed by the decrease in IFP, may reflect the impairment of tumour microcirculation subsequent to PDT.

The skillful help of Beatrice Sonntag and Christine Csapo and the critical and helpful comments of Drs Yves Boucher, Leo E. Gerweck, Intae Lee and Fan Yuan on the manuscript are gratefully acknowledged.

This study was supported by a grant from the Bundesministerium für Forschung und Technologie (No. 0706903A5) to A.E. Goetz and grants from the National Cancer Institute (CA-56591) to R.K. Jain. M. Leunig is recipient of a Feodor-Lynen Fellowship (1991–93) of the Humboldt Foundation, and R.K. Jain was the recipient of a Humboldt Senior Scientist Award (1990–91).

Abbreviations: PDT, photodynamic therapy; IFP, interstitial fluid pressure; MAP, mean arterial pressure.

### References

ASAISHI, K., ENDRICH, B., GOETZ, A. & MESSMER, K. (1981). Quantitative analysis of microvascular structure and function in the amelanotic melanoma A-Mel-3. *Cancer Res.*, **41**, 1898–1904.

BOUCHER, Y. & JAIN, R.K. (1992). Microvascular pressure is the principal driving force for interstitial hypertension in solid tumors: implications for vascular collapse. *Cancer Res.*, **52**, 5110–5114.

DAVIS, F.E., KENYON, K. & KIRK, J. (1953). A rapid titrimetric method for determining the water content of human blood. *Science*, **118**, 276–277.

DELLIAN, M., RICHERT, C., GAMARRA, F. & GOETZ, A.E. (1992). Tumor growth following PDT with functionalized porphycenes and photofrin II. In *Photodynamic Therapy and Biomedical Lasers*, Spinelli, P., Dal Fante, M. & Marchesini, R. (eds) pp. 467–469. Elsevier: Amsterdam.

FADNES, H.O., REED, R.K. & AUKLAND, K. (1977). Interstitial fluid pressure in rats measured with a modified wick technique. *Microvasc. Res.*, **14**, 27–36.

FINGAR, V.H., WIEMAN, T.J. & DOAK, K.W. (1991). Changes in tumor interstitial pressure induced by photodynamic therapy. *Photochem. Photobiol.*, **53**, 763–768.

FOSTER, T.H., PRIMAVERA, M.C., MARDER, V.J., HILF, R. & SPORN, L.A. (1991). Photosensitized release of von Willebrand Factor from cultured human endothelial cells. *Cancer Res.*, **51**, 3261–3266.

GERLOWSKI, L.E. & JAIN, R.K. (1986). Microvascular permeability of normal and neoplastic tissue. *Microvasc. Res.*, **31**, 288–305.

GULLINO, P.M. (1975). Extracellular compartment of solid tumors. In *Cancer*, Vol. 3, Becker, F.F. (ed.) pp. 327–354. Plenum: New York.

GUTMANN, R., LEUNIG, M., FEYH, J., GOETZ, A.E., KASTENBAUER, E., MESSMER, K. & JAIN, R.K. (1992). Interstitial hypertension in head and neck tumors in patients: correlation with tumor size. *Cancer Res.*, **52**, 1993–1995.

HENDERSON, B.W., WALDOW, S.M., MANG, T.S., POTTER, W.R., MALONE, P.B. & DOUGHERTY, T.J. (1985). Tumor destruction and kinetics of tumor cell death in two experimental mouse tumors following photodynamic therapy. *Cancer Res.*, **45**, 572–576.

JAIN, R.K. (1987). Transport of molecules in the tumor interstitium: a review. *Cancer Res.*, **47**, 3039–3051.

JAIN, R.K. (1988). Determinants of tumor blood flow: a review. *Cancer Res.*, **48**, 2641–2658.

LEE, I., BOUCHER, Y. & JAIN, R.K. (1992). Nicotinamide can lower tumor interstitial fluid pressure: mechanistic and therapeutic implications. *Cancer Res.*, **52**, 3237–3240.

LEUNIG, M., GOETZ, A.E., DELLIAN, M., ZETTERER, G., GAMARRA, F., JAIN, R.K. & MESSMER, K. (1992). Interstitial fluid pressure in solid tumors following hyperthermia: possible correlation with therapeutic response. *Cancer Res.*, **52**, 487–490.

LEUNIG, M., RICHERT, C., GAMARRA, F., LUMPER, W., VOGEL, E., JOCHAM, D. & GOETZ, A.E. (1993). Tumor localization kinetics of photofrin and three synthetic porphyrinoids in an amelanotic melanoma of the hamster. *Br. J. Cancer*, **68**, 225–234.

REED, M.W.R., MILLER, F.N., WIEMAN, T.J., TSENG, M.T. & PIETSCH, C.G. (1988). The effect of photodynamic therapy on the microcirculation. *J. Surg. Res.*, **45**, 452–459.

ROH, H.D., BOUCHER, Y., KALNICKI, S., BUCHSBAUM, R., BLOOMER, W.D. & JAIN, R.K. (1991). Interstitial hypertension in carcinoma of uterine cervix in patients: possible correlation with tumor oxygenation and therapeutic response. *Cancer Res.*, **51**, 6695-6698.

STAR, W.M., MARIJNISSEN, H.P.A., VAN-DEN-BERG-BLOK, A.E., VERSTEEG, J.A.C., FRANKEN, K.A.P. & REINHOLD, H.S. (1986). Destruction of rat mammary tumor and normal tissue microcirculation by hematoporphyrin derivative photoradiation observed *in vivo* in sandwich observation chambers. *Cancer Res.*, **46**, 2532–2540.

TRALAU, C.J., YOUNG, A.R., WALKER, N.P.J., VERNON, D.I., MACROBERT, A.J., BROWN, S.B. & BOWN, S.G. (1989). Mouse skin photosensitivity with dehaematoporphyrin ether (DHE) and aluminium sulphonated phthalocyanine (AlSPc): a comparative study. *Photochem. Photobiol.*, **49**, 305–312.

WEISS, N., DELIUS, M., GAMBIHLER, S., DIRSCHEDL, P., GOETZ, A.E. & BRENDEL, W. (1990). Influence of the shock wave application mode on the growth of A-Mel-3 and SSK2 tumors *in vivo*. *Ultrasound Med. Biol.*, **16**, 595–605.

WHITE, L., GOMER, C.J., DOIRON, D.R. & SZIRTH, B.C. (1988). Ineffective photodynamic therapy (PDT) in a poorly vascularized xenograft model. *Br. J. Cancer*, **57**, 455–458.

WIEMAN, T.J., MANG, T.S., FINGAR, V.H., HILL, T.G., REED, M.W.R., COREY, T.S., NGUYEN, V.Q. & RENDER, E.R. (1988). Effect of photodynamic therapy on blood flow in normal and tumor vessels. *Surgery*, **104**, 512–517.

WIIG, H. & GADEHOLD, G. (1985). Interstitial fluid pressure and hemodynamics in a sarcoma implanted in the rat tail. *Microvasc. Res.*, **29**, 176–189.

YUAN, F., LEUNIG, M., BERK, D.A. & JAIN, R.K. (1993). Microvascular permeability of albumin, vascular surface area and vascular volume measured in human adenocarcinoma LS174T using dorsal chamber in SCID mice. *Microvasc. Res.*, **45**, 269–289.

ZLOTECKI, R.A., BOUCHER, Y., LEE, I., BAXTER, L.T. & JAIN, R.K. (1993). Effect of angiotensin II induced hypertension on tumor blood flow and interstitial fluid pressure. *Cancer Res.*, **53**, 2466–2469.